# Association between vitamin D deficiency and liver fibrosis assessed by transient elastography in patients with metabolic dysfunction-associated steatotic liver disease

**DOI:** 10.1186/s12902-026-02527-0

**Published:** 2026-08-31

**Authors:** Justin Jose, Salman Muhammad Soomar, Ximena Fernandez Cristobal, Zahra Khan

**Affiliations:** 1 King’s College Hospital London, Dubai, United Arab Emirates; 2 King’s College London, London, UK; 3 Aga Khan University, Karachi, Pakistan

**Keywords:** MASLD, Vitamin D, Liver fibrosis, Transient elastography, FibroScan, Metabolic dysfunction

## Abstract

**Background:**

Metabolic dysfunction–associated steatotic liver disease is the most common chronic liver disease worldwide, affecting approximately 25–30% of adults. Liver fibrosis is the principal determinant of disease progression and mortality in MASLD. Vitamin D deficiency is highly prevalent in individuals with metabolic disorders and has been implicated in hepatic inflammation and fibrogenesis. However, the relationship between vitamin D status and liver fibrosis in MASLD remains incompletely understood, particularly in Middle Eastern populations.

**Methods:**

A retrospective cross‑sectional study was conducted at King’s College Hospital London, Dubai. Adult patients with MASLD who underwent vibration‑controlled transient elastography (FibroScan^®^) and had serum 25‑hydroxyvitamin D [25(OH)D] measurements within 90 days were included. Liver fibrosis was assessed using FibroScan-derived liver stiffness measurement. Hepatic steatosis was evaluated using both FibroScan-derived controlled attenuation parameter measurements and abdominal ultrasound findings where available. CAP was used as the quantitative measure of steatosis, while ultrasound grades were recorded for routine clinical assessment. Associations were evaluated using chi-square and non-parametric tests as appropriate. Multivariable logistic regression was performed to assess the independent association between vitamin D deficiency and clinically significant fibrosis.

**Results:**

A total of 301 patients with MASLD were included (mean age 41.75 ± 8.90 years; 50.5% male). Vitamin D deficiency was observed in 69.76% of patients. Most patients had no to mild fibrosis (F0–F1, 80.83%), followed by significant fibrosis (F2, 14.98%), advanced fibrosis (F3, 3.48%), and cirrhosis (F4, 0.69%). Vitamin D deficiency was significantly associated with fibrosis stage (*p* = 0.01) and hepatic steatosis severity (*p* = 0.01). No significant associations were observed between vitamin D deficiency and BMI (*p* = 0.20), diabetes/prediabetes (*p* = 0.69), hypertension (*p* = 0.92), or dyslipidaemia (*p* = 0.71), while a significant association was noted with metabolic syndrome (*p* < 0.01). Vitamin D deficiency was not independently associated with clinically significant fibrosis (aOR 0.89, 95% CI 0.44–1.80; *p* = 0.754). Increasing age (aOR 1.05, 95% CI 1.01–1.09; *p* = 0.018) and male sex (aOR 6.23, 95% CI 2.88–13.49; *p* < 0.001) were independently associated with clinically significant fibrosis.

**Conclusion:**

Vitamin D deficiency is highly prevalent in this MASLD cohort and is significantly associated with greater fibrosis severity, suggesting that it may serve as a marker of disease burden. However, causality cannot be established, and prospective studies are required to determine whether vitamin D plays a causal role in disease progression.

**Clinical trial number:**

Not applicable.

## Introduction

Metabolically–dysfunction–associated steatotic liver disease (MASLD), previously termed non‑alcoholic hepatic steatosis disease (NAFLD), is currently the most prevalent chronic liver disorder worldwide, affecting approximately 25–30% of adults globally and an even higher proportion in populations with a high burden of metabolic disease [1, 2]. Recent epidemiological analyses continue to underscore the rising global burden of MASLD, highlighting its close association with the increasing prevalence of obesity, type 2 diabetes, and other cardiometabolic risk factors [2]. MASLD represents a spectrum ranging from isolated hepatic steatosis to metabolic dysfunction–associated steatohepatitis (MASH), which can progress to advanced fibrosis, cirrhosis, and hepatocellular carcinoma (HCC) [2, 3]. According to recent consensus criteria, MASLD is diagnosed by the presence of hepatic steatosis in association with at least one cardiometabolic risk factor, such as overweight or obesity, type 2 diabetes mellitus, dyslipidaemia, or metabolic syndrome [4].

Among patients with MASLD, liver fibrosis has emerged as the most important determinant of long‑term clinical outcomes [5]. Longitudinal cohort data have demonstrated that fibrosis stage is a stronger predictor of both all‑cause and liver‑related mortality than the presence of steatohepatitis alone [5]. A large systematic review and meta‑analysis further confirmed an exponential increase in liver‑related mortality with advancing fibrosis stage, from minimal risk in stage F0 to markedly elevated risk in cirrhosis (F4) [6].

The role of vibration-controlled transient elastography (VCTE; FibroScan^®^) has expanded substantially in the MASLD era due to the increasing global burden of metabolic liver disease and the need for non-invasive fibrosis assessment. In contemporary hepatology practice, MASLD has become one of the leading indications for FibroScan referral, reflecting the growing clinical emphasis on fibrosis risk stratification and early identification of patients at increased risk of liver-related outcomes. Consequently, FibroScan has become an important component of routine evaluation and longitudinal monitoring in patients with MASLD [5, 6].

Consequently, accurate assessment of liver fibrosis is central to risk stratification and clinical management in MASLD. In this context, non‑invasive tools, particularly vibration‑controlled transient elastography (VCTE; FibroScan^®^), have become widely adopted for the routine assessment and longitudinal monitoring of liver fibrosis [7].

Given the central role of fibrogenesis in MASLD progression, increasing attention has been directed towards modifiable factors that may influence hepatic fibrosis. Vitamin D deficiency has been consistently associated with MASLD prevalence and disease severity [8]. Observational studies indicate that lower circulating 25‑hydroxyvitamin D [25(OH)D] concentrations are linked to more advanced steatosis and fibrosis among individuals with hepatic steatosis disease [9]. Experimental studies provide biological plausibility for this association, demonstrating that vitamin D exerts anti‑fibrotic effects through vitamin D receptor–mediated inhibition of hepatic stellate cell activation and suppression of profibrogenic gene expression [10].

Large population‑based analyses have strengthened the evidence linking vitamin D status to liver fibrosis. In a cross‑sectional analysis of the U.S. National Health and Nutrition Examination Survey (NHANES 2017–2018), individuals with optimal serum 25(OH)D concentrations (≥ 75 nmol/L) had significantly lower odds of both MASLD, and clinically significant fibrosis compared with those who were vitamin D deficient [11]. Similarly, among adults with MASLD, vitamin D sufficiency was associated with a substantially reduced risk of liver fibrosis, with evidence of a dose–response relationship across increasing vitamin D levels [12]. Comparable inverse associations have been reported in Asian cohorts, including studies demonstrating lower odds of advanced fibrosis among individuals with higher serum vitamin D concentrations, particularly in non‑obese and younger subgroups [13].

Interventional evidence, although limited, further supports a potential role for vitamin D in modifying liver disease severity. A single‑centre clinical trial from Egypt showed that six months of high‑dose intramuscular cholecalciferol supplementation led to significant improvements in FibroScan‑derived liver stiffness and controlled attenuation parameter scores in patients with NAFLD, alongside normalization of vitamin D levels [14]. However, these findings should be interpreted with caution due to small sample sizes, short follow‑up durations, and limited randomized evidence.

Regionally, the burden of MASLD is disproportionately high in the Middle East and Gulf Cooperation Council (GCC) countries, where prevalence estimates frequently exceed 40%, surpassing global averages [2, 15]. In the United Arab Emirates (UAE), metabolic risk factors are highly prevalent, with approximately three‑quarters of adults classified as overweight and nearly one‑sixth living with type 2 diabetes mellitus [16]. In parallel, vitamin D deficiency remains endemic despite abundant sunlight, with population‑based studies and systematic reviews reporting deficiency rates exceeding 60–70% among adults in the UAE [17, 18]. The coexistence of a high MASLD burden and widespread vitamin D deficiency underscores the importance of investigating the relationship between vitamin D status and liver fibrosis progression within this regional context.

## Methods

### Study design

This was a retrospective observational study conducted to evaluate the association between vitamin D status and liver fibrosis among adults with metabolic dysfunction–associated steatotic liver disease. The study is reported in accordance with Strengthening the Reporting of Observational studies in Epidemiology (STROBE) guidelines.

### Study setting

The study was conducted at King’s College Hospital London, Dubai, a tertiary care hospital with a capacity of approximately 100 beds and a broad range of medical and surgical specialties. The institution is the first hospital in the United Arab Emirates to establish a liver transplant program and serves a diverse, multicultural patient population.

### Study population and patient identification

Adult patients (aged ≥ 18 years) with a diagnosis of MASLD who underwent vibration-controlled transient elastography (FibroScan^®^) and had serum 25-hydroxyvitamin D [25(OH)D] levels measured during routine clinical evaluation between January and November 2025 were identified through the hospital’s electronic medical record system.

MASLD diagnosis was established using evidence of steatosis from either CAP measurements obtained during FibroScan or abdominal ultrasound examinations. MASLD was defined in accordance with current multisociety consensus criteria, requiring evidence of hepatic steatosis along with at least one cardiometabolic risk factor, including overweight/obesity, type 2 diabetes mellitus, prediabetes, hypertension, dyslipidaemia, or metabolic syndrome, and after exclusion of other causes of liver disease [13]. Insulin resistance alone was not considered an independent diagnostic criterion but was recorded separately when available.

### Eligibility criteria

#### Inclusion criteria

Participants were eligible if they met all of the following conditions:

- Age ≥ 18 years.
- Diagnosis of MASLD based on clinical, laboratory, and imaging criteria.
- Availability of a valid FibroScan examination with both controlled attenuation parameter (CAP) and liver stiffness measurement(LSM).
- Serum 25(OH)D measurement performed within 90 days of the FibroScan assessment.
- Complete demographic and anthropometric data available in the medical records.

#### Exclusion criteria

Patients were excluded if they had:

- Excessive alcohol consumption (> 20 g/day for women and > 30 g/day for men).
- Chronic viral hepatitis (hepatitis B or C).
- Autoimmune liver disease.
- Hereditary liver disorders (hemochromatosis, Wilson’s disease, or alpha-1 antitrypsin deficiency).
- Drug-induced liver injury.
- Advanced chronic kidney disease (stage 4 or 5; estimated glomerular filtration rate < 30 mL/min/1.73 m²).
- Active malignancy or life-limiting illness.
- Pregnancy.
- Malabsorptive disorders affecting vitamin D metabolism.
- Initiation of high-dose vitamin D supplementation (> 1000 IU/day) within 3 months prior to assessment.

### Sampling method and sample size

A purposive sampling approach was employed. All patient records meeting the eligibility criteria within the study period were included. A total of 301 patients fulfilled the inclusion criteria and were analysed.

### Data collection

Data were extracted retrospectively from electronic medical records using a standardized data abstraction form. Collected variables included:

- **Demographic and anthropometric data**: age, sex, nationality/ethnicity, body mass index (BMI).
- **Clinical history and comorbidities**: prediabetes, type 2 diabetes mellitus, hypertension, dyslipidaemia, obesity, and metabolic syndrome.
- **Laboratory parameters**: serum 25(OH)D, fasting plasma glucose, hemoglobin A1c (HbA1c), lipid profile (total cholesterol, LDL-cholesterol, HDL-cholesterol, triglycerides), liver enzymes (ALT, AST, GGT, alkaline phosphatase), albumin, platelet count, serum creatinine, and estimated glomerular filtration rate.
- **Imaging and non-invasive assessments**: FibroScan-derived CAP and LSM values, and abdominal ultrasound findings when available.

### Operational definitions

#### Vitamin D measurement and classification

Serum 25(OH)D levels were measured using a standardized chemiluminescent immunoassay on the Abbott Architect platform. Vitamin D status was classified according to widely accepted clinical thresholds:

- Deficiency: <50 nmol/L (< 20 ng/mL).
- Insufficiency: 50–74.9 nmol/L (20–29.9 ng/mL).
- Sufficiency: ≥75 nmol/L (≥ 30 ng/mL).

Primary analyses evaluated vitamin D deficiency (< 50 nmol/L) as the exposure variable, whereas continuous vitamin D concentrations were analysed secondarily. These thresholds were defined according to Endocrine Society Clinical Practice Guideline recommendations.

#### FibroScan examination and interpretation

Vibration-controlled transient elastography was performed using the FibroScan^®^ 502 Touch device (Echosens, Paris, France) by trained operators following standardized manufacturer protocols. The M probe was used for patients with BMI < 30 kg/m², and the XL probe for patients with BMI ≥ 30 kg/m² or when M-probe measurements were unreliable. Examinations were conducted after a minimum of 3 h of fasting.

A valid FibroScan examination required at least 10 valid measurements and an interquartile range-to-median ratio ≤ 30%. Median values were recorded for both CAP and LSM. Although a success rate ≥ 60% was documented according to institutional practice during the study period, reliability assessment primarily relied on contemporarily accepted measurement quality indicators.

FibroScan examinations were performed by trained operators experienced in vibration-controlled transient elastography according to institutional protocols.

### Hepatic steatosis and fibrosis classification

Hepatic steatosis severity and fibrosis stage were defined using previously validated VCTE cut-off values from published literature [13, 15].

#### Hepatic steatosis severity (CAP-based)

- S0 (no steatosis): <274 dB/m.
- S1 (mild): 274–289 dB/m.
- S2 (moderate): 290–301 dB/m.
- S3 (severe): ≥302 dB/m.

#### Fibrosis staging (LSM-based)

- F0–F1 (no to mild fibrosis): ≤8.1 kPa.
- F2 (significant fibrosis): 8.2–9.6 kPa.
- F3 (advanced fibrosis): 9.7–13.5 kPa.
- F4 (cirrhosis): ≥13.6 kPa.

These thresholds were selected based on prior validation studies in MASLD/NAFLD populations and are consistent with commonly used clinical cut-offs [13, 15].

### Metabolic syndrome

Metabolic syndrome was defined according to the International Diabetes Federation (IDF) criteria.

### Statistical analysis

The extent of missing data for each variable was assessed. Analyses were performed using complete-case methodology. No imputation procedures were undertaken. Continuous variables were summarized using means and standard deviations for normally distributed data, while categorical variables were expressed as frequencies and percentages. Participants were stratified based on vitamin D deficiency status. Associations between vitamin D status and clinical variables, including fibrosis stage and degree of hepatic steatosis, were assessed using the chi-square test or appropriate parametric/non-parametric tests as applicable. Because of sparse cell counts in advanced fibrosis categories, Fisher’s exact test was used when expected cell counts were < 5. To account for potential confounding, multivariable logistic regression was performed with clinically significant fibrosis (F2-F4) as the dependent variable. Covariates included age, sex, BMI, diabetes mellitus, hypertension, dyslipidaemia and metabolic syndrome. Adjusted odds ratios (aORs) with 95% confidence intervals were reported. A two-sided p-value ≤ 0.05 was considered statistically significant. Statistical analyses were performed using R statistical software.

## Results

Among the 301 patients, the cohort comprised 152 (50.50%) males and 149 (49.50%) females. The mean age ± SD in years was 41.75 ± 8.90. The majority of patients were from Europe and Central Asia (184 patients, 61.54%) (Table 1).

**Table 1:** Baseline characteristics of participants (*n* = 301)

Characteristics	n (%)
Age (Mean ± SD)	41.75± 8.90
Gender	
Male	152 (50.50)
Female	149 (49.50)
Ethnicity	
Europe and Central Asia	184 (61.54)
East Asia and Pacific	52 (17.39)
Middle East and Africa	44 (14.72)
Latin America and Caribbean	9 (3.01)
North America	10 (3.34)

The mean BMI ± SD was 30.80 ± 5.79, with 149 patients (50.16%) classified as obese (BMI ≥ 30). Metabolic comorbidities were frequent, including diabetes in 238 patients (79.07%), dyslipidemia in 208 (69.10%), and hypertension in 59 (19.60%). In addition, vitamin D deficiency was observed in 210 patients (69.76%). Regarding liver findings, hepatic steatosis on ultrasound was identified in 208 patients (69.10%), with Grade 1 being the most common (139 patients, 46.18%), followed by Grade 2 (58 patients, 19.27%), with smaller proportions in Grades 1–2 (7 patients, 2.33%), 2–3 (1 patient, 0.33%), and 3 (3 patients, 1.00%). Similarly, fibrosis staging by transient elastography showed that most patients had early-stage fibrosis, with F0–F1 in 232 patients (80.83%), followed by F2 in 43 (14.98%), F3 in 10 (3.48%), and F4 in 2 patients (0.69%) (Table 2).

**Table 2:** Clinical characteristics of patients (*n* = 301)

Characteristics	n (%)
BMI (Mean ± SD)	30.80 ± 5.79
BMI <30	148 (49.83)
BMI ≥30	149 (50.16)
Hypertension	
Yes	59 (19.60)
No	242 (80.40)
Diabetes	
Yes	238 (79.07)
No	63 (20.93)
Dyslipidaemia	
Yes	208 (69.10)
No	93 (30.90)
Vitamin D Deficiency	
Yes	210 (69.76)
No	91 (30.23)
Vitamin D level (ng/mL)	
(Mean ± SD)	29.00 ± 13.22
Fatty Liver	
Grade 1	139 (46.18)
Grade 1 to 2	7 (2.33)
Grade 2	58 (19.27)
Grade 2 to 3	1 (0.33)
Grade 3	3 (1.00)
Fib Level	
F0:F1	232 (80.83)
F2	43 (14.98)
F3	10(3.48)
F4	2 (0.69)

Patients with vitamin D deficiency had significantly lower serum vitamin D levels compared with non-deficient individuals (27.5 ± 11.6 vs. 50.3 ± 17.0 ng/mL). A significant association was observed between vitamin D deficiency and fibrosis stage (*p* = 0.01). For F0–F1 fibrosis, 166 patients (55.15%) were vitamin D deficient compared with 66 (21.93%) who were vitamin D sufficient. For F2 fibrosis, 27 patients (8.97%) were vitamin D deficient versus 16 (5.32%) vitamin D sufficient. Furthermore, all 10 F3 cases occurred in the vitamin D deficient group; however, this finding should be interpreted cautiously because of the small number of patients classified as F3 fibrosis (10, 3.32%), while F4 was observed in one patient in each group. Similarly, a significant association was observed for hepatic steatosis severity (*p* = 0.01). Among patients with vitamin D deficiency, Grade 1 hepatic steatosis was observed in 100 patients (33.22%) and Grade 2 in 47 (15.61%). In comparison, among vitamin D sufficient patients, Grade 1 was observed in 39 (12.96%) and Grade 2 in 11 (3.65%).

In contrast, no significant association was observed between vitamin D status and BMI (*p* = 0.20). However, vitamin D deficiency was significantly associated with metabolic syndrome (*p* < 0.001). No significant associations were found with diabetes/prediabetes/insulin resistance (*p* = 0.69), hypertension (*p* = 0.92), or dyslipidemia (*p* = 0.71). Ultrasound-defined steatosis severity was significantly associated with vitamin D deficiency (*p* = 0.01), whereas CAP-defined steatosis severity was not significantly associated with vitamin D status (*p* = 0.79) (Table 3).

**Table 3:** Association between Vitamin D deficiency and independent and clinical characteristics

Characteristics	Vitamin D Deficiency		*P*-value
Mean ± SD	Vitamin D Deficient (*n* = 210) 27.53 ± 11.60	Vitamin D Non-Deficient (*n* = 91) 50.29 ± 16.96	
BMI	30.50 ± 5.71	31.50 ± 5.94	0.20
Fatty Liver Grade			0.01
Fatty Liver	53 (17.61)	40 (13.29)	
Grade 1	100 (33.22)	39 (12.96)	
Grade 1 to 2	7 (2.33)	0 (0)	
Grade 2	47 (15.61)	11 (3.65)	
Grade 2 to 3	1 (0.33)	0 (0)	
Grade 3	2 (0.66)	1 (0.33)	
Fibrosis Level			0.01
F0:F1	166 (55.15)	66 (21.93)	
F2	27 (8.97)	16 (5.32)	
F3	10 (3.32)	0 (0)	
F4	1 (0.33)	1 (0.33)	
Diabetes / Prediabetes/ Insulin Resistance			0.69
Present	164 (54.67)	74 (24.67)	
Not Present	45 (15)	17 (5.67)	
Hypertension			0.92
Present	42 (13.95)	17 (5.65)	
Not Present	168 (55.81)	74 (24.58)	
Metabolic Syndrome			0.00
Present	9 (2.99)	13 (4.32)	
Not Present	201 (66.78)	78 (25.91)	
Dyslipidemia			0.71
Present	147 (48.84)	61 (20.27)	
Not Present	63 (20.93)	30 (9.97)	

Data are presented as n (%) within each vitamin D category. Percentages represent column percentages unless otherwise specified

Across fibrosis stages, mean vitamin D levels were 28.5 ng/mL in F0-F1, 31.6 ng/mL in F2, 23.9 ng/mL in F3, and 20.35 ng/mL in F4, suggesting lower vitamin D levels in advanced fibrosis (Table 4).

**Table 4:** Serum vitamin D levels across fibrosis stages

Fibrosis Stage	Vitamin D level (ng/mL), Mean ± SD
F0-F1	28.49 ± 11.30
F2	31.56 ± 18.08
F3	23.90 ± 10.25
F4	20.35 ± 13.72

**Table 5:** Multivariable logistic regression analysis reporting adjusted odds ratio (aOR) for clinically significant fibrosis (F2-F4)

Characteristics	Adjusted OR	95% CI	*p*-value
Vitamin D deficiency	0.89	0.44–1.80	0.754
Age (per year increase)	1.05	1.01–1.09	0.018
Male sex	6.23	2.88–13.49	< 0.001
BMI (kg/m²)	1.03	0.98–1.09	0.276
Diabetes/prediabetes/insulin resistance	0.89	0.40–2.01	0.782
Hypertension	1.08	0.51–2.29	0.851
Metabolic syndrome	1.75	0.56–5.45	0.332
Dyslipidemia	1.90	0.86–4.20	0.113

Multivariable logistic regression demonstrated that vitamin D deficiency was not independently associated with clinically significant fibrosis (aOR 0.89, 95% CI 0.44–1.80; *p* = 0.754). Increasing age (aOR 1.05, 95% CI 1.01–1.09; *p* = 0.018) and male sex (aOR 6.23, 95% CI 2.88–13.49; *p* < 0.001) were independently associated with clinically significant fibrosis. No significant associations were observed for BMI, diabetes/prediabetes/insulin resistance, hypertension, metabolic syndrome, or dyslipidaemia (Table 5).

## Discussion

In this study, we observed a high prevalence of vitamin D deficiency among patients undergoing routine vibration-controlled transient elastography for metabolic dysfunction-associated steatotic liver disease. Nearly 70% of patients demonstrated deficient serum 25-hydroxyvitamin D levels, reflecting a substantial burden of hypovitaminosis D within this metabolically high-risk cohort. However, this prevalence is broadly consistent with previously reported estimates in the general population of the United Arab Emirates and other regional cohorts, suggesting that the observed rates may reflect underlying population-level trends rather than a disease-specific excess [19, 20].

A key finding of this study is the significant association between vitamin D deficiency and advanced liver fibrosis in unadjusted analyses. Patients with lower 25(OH)D concentrations exhibited higher fibrosis stages on transient elastography, with all individuals classified as stage F3 being vitamin D deficient. However, this observation should be interpreted cautiously given the small number of patients with advanced fibrosis. Furthermore, multivariable analysis demonstrated that vitamin D deficiency was not independently associated with clinically significant fibrosis after adjustment for relevant covariates, suggesting that the observed relationship may be influenced by underlying demographic and metabolic factors. These findings are consistent with prior observational studies. Ko et al. reported that vitamin D deficiency was independently associated with advanced liver fibrosis assessed by transient elastography in patients with chronic liver disease, with more than a three-fold increase in the risk of severe fibrosis [21]. Similarly, Behera et al. demonstrated that patients with chronic hepatitis C and vitamin D deficiency had significantly higher METAVIR fibrosis scores, identifying serum vitamin D as an independent negative predictor of fibrosis severity [22].

Population-based data further support our observations. Using NHANES 2017–2018 data, Ji and Gu demonstrated that vitamin D sufficiency was associated with significantly lower odds of liver fibrosis, with a clear dose-dependent relationship across increasing quartiles of serum 25(OH)D levels [23]. In our cohort, ultrasound-defined steatosis severity was significantly associated with vitamin D deficiency, whereas CAP-defined steatosis severity did not demonstrate a statistically significant association. Differences between ultrasound- and CAP-based assessments may reflect variation in measurement techniques, grading systems, and sensitivity to hepatic fat accumulation. The discrepancy between our findings and those reported in NHANES may also relate to differences in study populations, obesity burden, metabolic risk profiles, and methods used to assess steatosis severity. Our cohort represented a tertiary-care MASLD population with a high prevalence of obesity and diabetes, which differs substantially from the population-based NHANES cohort [23].

Comparable associations have been observed in other chronic liver diseases. In a cohort of patients with chronic hepatitis B, Hashemi et al. reported significantly higher liver stiffness measurements among vitamin D-deficient individuals, reinforcing the relationship between low vitamin D status and hepatic fibrosis independent of disease aetiology [24]. Differences in the magnitude of association between MASLD and viral hepatitis populations may reflect variations in inflammatory activity, fibrosis kinetics, and disease pathophysiology.

Although the retrospective nature of our study precludes causal inference, interventional studies provide supportive evidence for a potential therapeutic role of vitamin D. In a randomized, double-blind, placebo-controlled trial, Lukenda Zanko et al. demonstrated that vitamin D supplementation led to significant reductions in liver stiffness over 6–12 months in patients with NAFLD [25]. Similarly, Gad et al. observed significant improvements in liver stiffness and controlled attenuation parameter following six months of high-dose cholecalciferol therapy [26]. These findings align with our observation that vitamin D deficiency was significantly associated with both advanced fibrosis and greater hepatic steatosis severity.

Mechanistic data provide biological plausibility for these associations. Hepatic stellate cells, the principal effector cells in liver fibrogenesis, express the vitamin D receptor, through which vitamin D signalling can directly modulate fibrogenic responses. Experimental studies have shown that activation of the vitamin D receptor suppresses hepatic stellate cell activation, proliferation, and collagen synthesis, key processes in fibrosis progression. Beilfuss et al. demonstrated that vitamin D counteracts transforming growth factor-β-mediated profibrotic signalling in human hepatic stellate cells, providing direct evidence for its antifibrotic effects [27]. In addition, vitamin D has been shown to attenuate hepatic inflammation and oxidative stress while modulating key fibrogenic pathways, including nuclear factor-κB and transforming growth factor signalling [28, 29]. These mechanisms provide biological plausibility for the observed association between vitamin D deficiency and liver fibrosis severity.

The absence of a significant association between vitamin D deficiency and diabetes/prediabetes may partly reflect the very high prevalence of diabetes within the study cohort (79%), creating a ceiling effect that limited discrimination between exposure groups. Similarly, the lack of association with BMI may be attributable to the relatively homogeneous BMI distribution, with a mean BMI of 30.8 kg/m² and a large proportion of participants classified as obese. These characteristics may have reduced statistical power to detect modest between-group differences. Selection bias inherent to a tertiary-care population undergoing FibroScan assessment may have further contributed to a relatively homogeneous metabolic risk profile. In addition, the cross-sectional design and timing of vitamin D measurement may not adequately capture longitudinal relationships between vitamin D status and glycaemic control.

The significant association between vitamin D deficiency and metabolic syndrome should also be interpreted cautiously because only a small number of patients met metabolic syndrome criteria. Furthermore, the absence of significant associations with individual metabolic components such as diabetes, hypertension, and dyslipidaemia suggests that this finding may be unstable and requires confirmation in larger cohorts.

The observed association between vitamin D deficiency and fibrosis severity should be interpreted alongside the results of the adjusted analyses. Although unadjusted analyses demonstrated a significant association between vitamin D deficiency and fibrosis stage, this relationship was not retained after adjustment for demographic and metabolic covariates. This finding suggests that age, sex, and other metabolic factors may contribute substantially to the observed relationship between vitamin D status and liver fibrosis. Nevertheless, the consistency of our findings with prior observational studies supports continued investigation of vitamin D as a potential marker of liver disease severity.

In addition, FibroScan-derived liver stiffness measurements may overestimate fibrosis severity in the presence of active hepatic inflammation or steatohepatitis. Although transient elastography is a validated non-invasive method, this potential limitation should be considered when interpreting fibrosis classifications. Serum 25(OH)D concentrations were measured between January and November 2025, and seasonal variation in sunlight exposure, outdoor activity, clothing habits, and behavioural adaptation to extreme climatic conditions may have influenced vitamin D concentrations. Consequently, some degree of exposure misclassification cannot be excluded. As a single-centre tertiary-care study, referral and selection bias may also limit the generalisability of our findings to broader MASLD populations.

Collectively, our findings add to a growing body of evidence linking vitamin D deficiency with increased liver fibrosis severity across diverse liver diseases. The consistency of findings across observational, population-based, mechanistic, and interventional studies supports a biologically meaningful relationship, while highlighting the need for further prospective and interventional research to clarify causality and therapeutic implications. Future prospective longitudinal studies are warranted to clarify the temporal relationship between vitamin D deficiency and fibrosis progression in MASLD. Randomized controlled trials evaluating liver-specific outcomes are needed to determine whether correction of vitamin D deficiency can modify disease progression.

### Strengths and limitations

This study has several strengths. It included a relatively large cohort of patients undergoing standardized vibration-controlled transient elastography (VCTE) assessment in routine clinical practice. The study provides insight into a diverse tertiary-care population in the United Arab Emirates, adding regional data from a population in which both MASLD and vitamin D deficiency are highly prevalent. Additionally, the use of transient elastography enabled non-invasive assessment of liver fibrosis across a broad spectrum of disease severity.

Several limitations should be acknowledged. Due to the retrospective cross-sectional study design, causal relationships between vitamin D deficiency and liver fibrosis cannot be established. Serum 25(OH)D concentrations were measured between January and November 2025, and seasonal variation in sunlight exposure, outdoor activity, and behavioural adaptation to extreme climatic conditions may have influenced vitamin D concentrations, introducing potential exposure misclassification. Liver fibrosis was assessed using transient elastography rather than liver biopsy, the histological gold standard; although VCTE is a validated and widely used non-invasive modality, liver stiffness measurements may be influenced by factors other than fibrosis, including hepatic inflammation, cholestasis, congestion, and other transient physiological conditions. FibroScan examinations were performed at a single time point, precluding assessment of fibrosis progression or regression over time. The small number of patients with advanced fibrosis (F3-F4) may have reduced statistical stability and limited the precision of fibrosis-stage analyses. As a single-centre tertiary referral study, the cohort may have had a greater burden of metabolic and liver disease than community-based MASLD populations, introducing referral and selection bias and limiting generalisability. In addition, complete data required for reliable assessment of at-risk MASH using the FAST score were unavailable, precluding evaluation of this clinically relevant outcome. Finally, although FibroScan examinations were performed by trained personnel using standardized protocols, transient elastography remains an operator-dependent technique and some degree of inter-operator variability cannot be excluded.

## Conclusion

Vitamin D deficiency is highly prevalent among patients with MASLD and is significantly associated with advanced liver fibrosis in this cohort. These findings are consistent with existing observational, population‑based, and mechanistic evidence suggesting a potential link between vitamin D status and hepatic fibrogenesis.

However, given the cross‑sectional and retrospective nature of this study, causal relationships cannot be established. Vitamin D deficiency may represent a potentially modifiable factor associated with fibrosis severity, but its role in disease progression remains uncertain. Further prospective and interventional studies are required to determine whether improving vitamin D status can influence the natural history of MASLD and improve liver‑related outcomes.

Although vitamin D status may represent a clinically relevant marker in patients with MASLD, routine use as a standalone fibrosis risk stratification tool cannot currently be recommended.

## Data Availability

Data and materials are available with the corresponding author on reasonable request.

## Abbreviations

25(OH)D: 25‑Hydroxyvitamin D
ALT: Alanine Aminotransferase
AST: Aspartate Aminotransferase
BMI: Body Mass Index
CAP: Controlled Attenuation Parameter
CLIA: Chemiluminescent Immunoassay
eGFR: Estimated Glomerular Filtration Rate
F0: F4–Liver Fibrosis Stages
GGT: Gamma‑Glutamyl Transferase
HbA1c: Hemoglobin A1c
HCC: Hepatocellular Carcinoma
HSCs: Hepatic Stellate Cells
IQR: Interquartile Range
JCIA: Joint Commission International Accreditation
LSM: Liver Stiffness Measurement
MASLD: Metabolic Dysfunction–Associated Steatotic Liver Disease
MASH: Metabolic Dysfunction–Associated Steatohepatitis
NAFLD: Non‑Alcoholic Hepatic steatosis Disease
NHANES: National Health and Nutrition Examination Survey
PTH: Parathyroid Hormone
SD: Standard Deviation
TE: Transient Elastography
UAE: United Arab Emirates
VCTE: Vibration‑Controlled Transient Elastography
VDR: Vitamin D Receptor
